# New Perspectives on the Adverse Effects of NSAIDs in Cancer Pain: An Italian Delphi Study from the Rational Use of Analgesics (RUA) Group †

**DOI:** 10.3390/jcm11247451

**Published:** 2022-12-15

**Authors:** Giustino Varrassi, Flaminia Coluzzi, Diego Fornasari, Flavio Fusco, Walter Gianni, Vittorio Andrea Guardamagna, Filomena Puntillo, Giovanni Sotgiu

**Affiliations:** 1 Giustino Varrassi, Paolo Procacci Foundation, Via Tacito 7, 00193 Rome, Italy; 2 Department of Medical and Surgical Sciences and Biotechnologies, Sapienza University of Rome, Polo Pontino, 04013 Latina, Italy; 3 Anesthesiology, Intensive Care, and Pain Medicine Unit, Sant’Andrea University Hospital, 00189 Rome, Italy; 4 Department of Medical Biotechnology and Molecular Medicine, Università degli Studi di Milano, 20121 Milan, Italy; 5 Palliative Care Unit, Department of Primary and Community Care, ASL3, 16100 Liguria, Italy; 6 IRCCS Santa Lucia Foundation, 00179 Rome, Italy; 7 Pain Medicine Unit, Istituto Europeo Oncologico, 20141 Milan, Italy; 8 Department of Interdisciplinary Medicine (DIM), University of Bari, 70124 Bari, Italy; 9 Anesthesia, Intensive Care and Pain Unit, Policlinico Hospital, 70124 Bari, Italy; 10 Clinical Epidemiology and Medical Statistics Unit, Department of Medicine, Surgery and Pharmacy, University of Sassari, 07100 Sassari, Italy

**Keywords:** cancer pain, NSAIDs, paracetamol, adverse events

## Abstract

Non-steroidal anti-inflammatory drugs (NSAIDs) are among the most frequently prescribed drugs for cancer pain. We used the Delphi methodology to evaluate the opinions of clinicians on NSAIDs and paracetamol, with a specific focus on their safety profile. Consensus was reached on seven statements. A high level of consensus was reached regarding the use of NSAIDs and gastrointestinal, cardiovascular, and renal risk in patients taking low-dose aspirin and assessment of liver function during long-term treatment with paracetamol. Consensus was also reached that assessment and monitoring of eGFR are important in the elderly being administered NSAIDs. It was further agreed that NSAIDs can often play a key role in association with opioids in the treatment of cancer pain and that paracetamol is the analgesic of first choice for patients with mild chronic pain. When NSAIDs are administered in combination with steroids, it was agreed that the risk of gastrointestinal damage is increased since steroids delay the healing of ulcers and that paracetamol can be used during pregnancy and does not affect the health of the fetus. This Delphi study highlights that there is poor agreement on how these drugs are routinely prescribed. However, a consensus was reached for seven key statements and may represent a valid contribution to daily practice.

## 1. Introduction

The vast majority of patients with cancer experience pain [1], and for many, it is often poorly managed [2]. The WHO analgesic ladder provides recommendations for the treatment of cancer pain, including a three-step approach based on analgesics, with nonopioids, and weak and strong opioids for mild, moderate, and severe pain, respectively [3]. In addition, the oral administration of drugs should be favored whenever possible.

Current guidelines from the European Society for Medical Oncology (ESMO) highlight that paracetamol, known for its analgesics but non-anti-inflammatory properties, and the non-steroidal anti-inflammatory drugs (NSAIDs) should be universally accepted [4]. Thus, non-opioid analgesics, with their different properties, are the possible options for the first two steps: actually, this means paracetamol and NSAIDs are the first step in pain management [4].

However, high-quality evidence to support paracetamol alone or in combination with opioids is missing [5]. Besides paracetamol, NSAIDs are among the most frequently prescribed drugs and are listed in the WHO’s Essential Medicines.

However, NSAIDs’ adverse events include gastrointestinal, cardiovascular, hepatic, renal, cerebral, and pulmonary complications [6]. The type and dose of NSAID should be individualized after adequate stratification of the gastrointestinal and cardiovascular risk in each patient [7]. Clinicians often prescribe NSAIDs despite the lack of evidence in certain settings [8]. In some specific cases, a gastroprotective agent is co-prescribed, although this approach is not standardized. Proton-pump inhibitors significantly reduce the risk of gastrointestinal bleeding in high-risk patients, such as those using antiplatelet agents or oral anticoagulant drugs [9]. On the other hand, COX-2 selective agents, although safer for the gastrointestinal tract, have a worse cardiovascular profile, and should be theoretically avoided in patients with a high risk of cardiovascular events and thrombosis, such as in cancer patients. However, the recent literature supports the hypothesis that COX-2 overexpression promotes tumor growth and that COX-2 inhibitors, as a combination strategy, may exert a therapeutic effect in cancer [10]. Furthermore, clinicians may have different perceptions of patients’ pain and management [4].

Despite current drugs’ dose-limiting toxic effects, pharmacotherapy remains an important component of pain management: combination drug regimens have been followed by clinicians to improve pain control [11]. Moreover, as novel cancer therapies increase survival, it becomes more and more important to maintain quality of life for long-term survivor patients, experiencing chronic pain [12].

Chronic cancer pain depends both on tumor and treatment, resulting in several pain sensations. Therefore, pain caused by tumor lesions should be described feasibly as nociceptive or neuropathic, or a mix of them [12]. There is no evidence of nonopioid drug combinations for any indication, while duloxetine, pregabalin, and gabapentin seem to give better results in controlling chronic pain [12].

Due to the lack of knowledge, the Rational Use of Analgesics (RUA) project is investigating the clinical management of cancer pain in Italy, which has found therapeutic challenges and prescription-related discrepancies between guidelines and routine clinical practice [13]. Furthermore, no consensus was found on the use of strong opioids [14]. Based on the Delphi methodology, we evaluated the opinions of attending physicians on analgesic, paracetamol, and anti-inflammatory, NSAIDs, in cancer pain management, with a specific focus on their safety profile.

## 2. Materials and Methods

### 2.1. Study Design

A series of virtual meetings (the last one in March 2022) were conducted. A two-round Delphi approach was adopted [13]. A steering committee was selected, including experts in pain medicine, pharmacology, medical statistics, and palliative care: 7 persons met virtually to define a list of statements to be voted upon with a focus on the safety profile of NSAIDs and paracetamol. The statements dealt with aspects that the steering committee considered controversial, or for which limited information is available in the scientific literature. 

### 2.2. Delphi Process

The Delphi process, which is an indirect, anonymous, iterative method aimed at achieving consensus among experts, is useful in situations where evidence is weak or missing [15,16]. Consensus can be achieved through a working group of experts using an interactive process of individual feedback. One hundred and sixty-three experts agreed to participate. The entire Delphi process was carried out virtually. In the first Delphi round, a brief overview of the project was provided. Each statement was presented, and voting was anonymously carried out. Using a 5-point Likert scale (0 = totally disagree and 4 = totally agree), consensus on a statement was achieved if the median consensus score (expressed as a value in which at least 50% of participants agreed) was at least 4 and the interquartile range (IQR) was 3–4. In the second Delphi round, statements for which consensus was not reached were revised by the steering committee based on feedback from the participants and voted upon during the successive Delphi session. According to national policies, Ethics Committee approval was not required.

### 2.3. Statistical Analysis

Qualitative variables were summarized with absolute and relative (percentage) frequencies, whereas quantitative variables were described with means (standard deviations) or medians (IQR) based on their parametric and non-parametric distribution, respectively. Chi-squared or Fisher exact tests were used to compare groups in relation to qualitative variables. A two-tailed p-value of less than 0.05 was considered statistically significant. The statistical software used for all statistical computations was STATA version 16 (StataCorp, Brazos, TX, USA).

## 3. Results

Panelists were representative of the entire national territory, and 141/163 participants were specialists: 29% were palliative care physicians, 24% were oncologists, and 19.5% worked in anesthesia, intensive care, and pain medicine. In addition, 39% had >20 years of experience in their field. Moreover, participants were mainly involved in home-based palliative care (56%) and outpatient clinics (32.7%). 

Twenty-one statements were formulated by the steering committee and voted upon. Consensus was reached for 7/21 statements: five in the first and two in the second round (Table 1). Regarding the use of NSAIDs in patients taking low-dose aspirin, consensus was not reached on the initial statement that almost all NSAIDs are contraindicated because they can increase the risk of gastrointestinal, cardiovascular, and renal events. However, a high level of consensus was reached when the statement was changed to “NSAIDs increase gastrointestinal, cardiovascular, and renal risk in patients taking low-dose aspirin”. It was also agreed that liver function must be monitored during long-term treatment with paracetamol and that assessment and monitoring of eGFR are important in the elderly when NSAIDs are prescribed. Moreover, the statement “NSAIDs can often play a key role in association with opioids in treatment of cancer pain” was confirmed. Participants reached a consensus that paracetamol is the analgesic of first choice for patients with mild chronic pain. When NSAIDs are administered in combination with steroids, the statement that the risk of gastrointestinal damage is increased since steroids delay the healing of ulcers (micro-ulcers) was approved. Lastly, consensus was reached on the statement “paracetamol can be used during pregnancy and does not affect the health of the unborn child”.

In contrast, consensus was not reached for the remaining 14 statements which covered a broad range of topics, such as pharmacological properties of NSAIDs, fixed-dose combinations, combination with anticoagulants, long-term administration, and safety (Table 2).

## 4. Discussion and Conclusions

The present work is an extension of a previous one, which achieved consensus on strong opioids and individualization of therapy in routine practice [14]. Herein, the overall aim was to achieve consensus on NSAID or non-opioid analgesic in patients with mild pain. Consensus was achieved for only 7 of the 21 statements. This highlights that there is poor agreement on how these drugs are routinely prescribed. 

In particular, disagreement was related to the role of NSAIDs in the increased risk of gastrointestinal, cardiovascular, and renal adverse events in patients taking low-dose aspirin. Several manuscripts have underscored this risk [17,18]. The risk of renal events may be associated with a higher risk of incident eGFR <60 mL/min per 1.73 m^2^ and eGFR decline ≥30%, although the risk can vary according to the NSAID [19]. The recent recommendations of APAGE/APLAR/APSDE/APSH/APSN/PoA on patients with hypertension or cardiovascular, renal, or gastrointestinal comorbidities emphasize individual assessment before prescription [20]. In patients with high cardiovascular risk, naproxen and celecoxib are the preferred drugs. As a general rule, the lowest effective NSAID dose should be administered for the shortest period of time, while preferring an immediate-release formulation [18]. In addition, concomitant use of an NSAID with corticosteroids, other antiplatelet/anticoagulation agents, or low-dose aspirin should be avoided [18].

There was broad consensus on monitoring liver function during long-term exposure to paracetamol. Recently, several cases of paracetamol-induced liver intoxication have been described worldwide [21], and the highest dosages may lead to irreversible acute liver failure [22]. In reality, paracetamol overdose is the only recognized cause of hepatotoxicity, whereas limited data are available regarding the potential toxicity of long-term use. Patients using paracetamol for chronic pain are 4-fold more likely to have slightly abnormal results on liver function tests and frail elderly patients may have reduced clearance of paracetamol glucuronide [23].

It was held that NSAIDs can play a key role in association with opioids in the treatment of cancer pain. NSAIDs are widely used to treat cancer pain and are frequently used in combination with opioids [24]. NSAIDs are particularly useful as co-analgesics in the management of metastatic bone pain and in advanced cancers, although the risk of long-term adverse effects should be quantified even in the cancer setting. Non-opioid analgesics are recommended for this purpose in the WHO treatment ladder, either alone or in combination with opioids. Notwithstanding, limited evidence is available on the combined prescription of NSAIDs and opioids [25]. 

Assessment and monitoring of eGFR are recommended in the elderly to select the most appropriate analgesic, with NSAIDs being associated with acute renal failure [26].

The PAGE/APLAR/APSDE/APSH/APSN/PoA includes the elderly in the category of ‘high-risk’ patients, and renal function should be monitored if not carried out in the past 6 months [20].

Paracetamol was also considered the first choice in mild chronic pain in agreement with the current literature [27]. In addition, a review of guideline recommendations found that the majority recommend paracetamol as first- or second-line for both acute and chronic pain [28]. It can be prescribed to patients with limited therapeutic options, for those with liver, kidney, or cardiovascular diseases, and for gastrointestinal disorders, as well as in the elderly [27,28]. Moreover, kidney disease has been shown to be highly prevalent in cancer patients. Therefore, the evaluation of creatinine clearance should drive the correct analgesic prescription and dosage adaptation [29].

Consensus was also reached on the statement related to the increased gastrointestinal risk when steroids are prescribed with NSAIDs, since steroids delay the healing of ulcers (micro-ulcers), as previously demonstrated [30]. More recently, it was reported that short-term administration of steroids may be associated with gastrointestinal bleeding, which is increased by the concomitant administration of NSAIDs [31]. In an analysis of over 2000 cases of upper gastrointestinal complications, steroids were associated with an increased risk of bleeding (OR 1.8), and concurrent use of steroids with low-medium and high doses of an NSAID was associated with ORs of 4.0 and 12.7, respectively [32]. 

The last statement achieving consensus was on the use of paracetamol during pregnancy and the safety of the unborn child. Historically, paracetamol has been considered safe for use in pregnancy [33,34]. However, caution on paracetamol during pregnancy has recently been advocated since it may increase the risk of neurodevelopmental, reproductive, and urogenital disorders [31]. It may also be associated with sleep and attention problems in children [35]. Given these concerns, the lowest effective dose for the shortest possible time should be administered [33]. 

The statements not reaching consensus covered several topics, including pharmacological interactions, mechanism of action and pharmacological properties, combination with oral anticoagulants, and choice of NSAID: this seems to underscore poor heterogeneity in routine practice. Clear recommendations, based on high-quality evidence, and improved education on NSAIDs are thus needed. This missing consensus can indeed be considered a study limitation. However, the consensus reached for seven key statements may represent a valid contribution to daily practice.

## Figures and Tables

**Table 1:** Statements for which consensus was reached.

STATEMENT	REVISED STATEMENT	SCORE GIVEN N (%)					MEDIAN (IQR)
		0	1	2	3	4	
In patients taking low-dose aspirin, almost all NSAIDs are contraindicated because they increase the risk of gastrointestinal, cardiovascular, and renal events	NSAIDs increase gastrointestinal, cardiovascular, and renal risk in patients taking low-dose aspirin	1 (0.78)	6 (4.69)	11 (8.59)	37 (28.91)	73 (57.03)	4 (3–4)
Liver function should be monitored during long-term treatment with paracetamol		0 (0.00)	0 (0.00)	5 (3.91)	34 (26.56)	89 (69.53)	4 (3–4)
NSAIDs can often play a key role in association with opioids in treatment of cancer pain		1 (0.78)	4 (3.13)	11 (8.59)	34 (26.56)	78 (60.94)	4 (3–4)
Assessment and monitoring of eGFR is recommended in the elderly to select the most appropriate analgesic	Assessment and monitoring of eGFR is important in the elderly when using NSAIDs	1 (0.78)	3 (2.34)	19 (14.84)	33 (25.78)	72 (56.25)	4 (3–4)
Paracetamol is the analgesic of first choice in mild chronic pain		4 (3.13)	5 (3.91)	12 (9.38)	28 (21.88)	79 (61.72)	4 (3–4)
The association of steroids with NSAIDs increases the gastrointestinal damage caused by the latter since steroids delay the healing of ulcers (micro-ulcers)		1 (0.78)	8 (6.25)	11 (8.59)	40 (31.25)	68 (53.13)	4 (3–4)
Paracetamol can be used during pregnancy and does not affect the health of the unborn child		0 (0.00)	4 (3.13)	12 (9.38)	43 (33.59)	69 (53.91)	4 (3–4)

**Table 2:** Statements for which consensus was not reached.

STATEMENT	REVISED STATEMENT	0	1	2	3	4	MEDIAN (IQR)
Paracetamol has no anti-inflammatory properties; its analgesic properties are not dependent on COX inhibition		8 (6.25)	11 (8.59)	13 (10.16)	29 (22.66)	67 (52.34)	4 (2.5–4)
All NSAIDs, selective, preferential, and traditional, especially at high doses, increase cardiovascular risk by inhibiting endothelial COX-2	Traditional NSAIDs and not just COX inhibitors inhibit endothelial COX2 and increase cardiovascular risk, especially at high doses	2 (1.56)	8 (6.25)	16 (12.50)	51 (39.84)	51 (39.84)	3 (3–4)
NSAIDs, like many other drugs, can cause a variety of adverse reactions due to individual genetic variance		2 (1.56)	8 (6.25)	19 (14.84)	45 (35.16)	54 (42.19)	3 (3–4)
NSAIDs, by inhibiting COX in inflamed tissues, counteract peripheral sensitization		4 (3.13)	6 (4.69)	20 (15.63)	44 (34.38)	54 (42.19)	3 (3–4)
It is always possible to prescribe acetaminophen in combination with oral anticoagulants		6 (4.69)	13 (10.16)	18 (14.06)	42 (32.81)	49 (38.28)	3 (2–4)
Fixed-dose combinations of paracetamol and other drugs are indicated in the treatment of acute pain or exacerbations of chronic pain	Fixed-dose combinations of paracetamol and opioids are indicated in treatment of acute pain or exacerbations of chronic pain	17 (13.28)	15 (11.72)	13 (10.16)	43 (33.59)	40 (31.25)	3 (1.5–4)
Metamizole has analgesic, antipyretic, and antispasmodic properties but not anti-inflammatory	Metamizole is an analgesic, antipyretic, and antispasmodic drug, with unclear anti-inflammatory properties	12 (9.38)	12 (9.38)	27 (21.09)	41 (32.03)	36 (28.13)	3 (2–4)
In patients with renal insufficiency, the use of paracetamol does not require dose adjustments	The daily dose of paracetamol does not need to be adjusted in patients with renal insufficiency	22 (17.19)	23 (17.97)	17 (13.28)	35 (27.34)	31 (24.22)	3 (1–3)
NSAIDs are not appropriate for the long-term treatment of chronic inflammatory pain, as they mainly act on peripheral inflammatory processes	NSAIDs act as peripheral anti-inflammatories and are not indicated for chronic pain	18 (14.06)	15 (11.72)	26 (20.31)	34 (26.56)	35 (27.34)	3 (1–4)
In the choice of NSAIDs, ibuprofen, diclofenac or ketorolac should be preferred to piroxicam, indomethacin, or naproxen in patients at risk of gastrointestinal adverse events.	Ibuprofen and diclofenac have lower gastrointestinal toxicity than piroxicam and indomethacin	15 (11.72)	16 (12.50)	15 (11.72)	43 (33.59)	39 (30.47)	3 (2–4)
Gastrointestinal toxicity from NSAIDs can be mitigated by intervening on intestinal microbiota	Intestinal microbiota can improve intestinal homeostasis by reducing the risk of NSAID-related enteropathy	10 (7.81)	7 (5.47)	22 (17.19)	43 (33.59)	46 (35.94)	3 (2–4)
Steroids do not cause extensive gastrointestinal damage, and at least in the short to medium term gastroprotection is not required	During steroid therapy, gastric protection is needed only for long-term treatment and not for short-term therapy	26 (20.31)	15 (11.72)	6 (4.69)	38 (29.69)	43 (33.59)	3 (1–4)
All SSRIs interact with NSAIDs, increasing their gastrointestinal risk	The gastrointestinal risk from NSAIDs is greater in patients taking SSRIs	16 (12.50)	21 (16.41)	29 (22.66)	32 (25.00)	30 (23.44)	2 (1–3)
Chronic use of paracetamol in patients taking salicylates is not recommended due to the risk of nephropathy	In patients taking salicylates, chronic use of high-dose paracetamol increases the risk of nephropathy	22 (17.19)	25 (19.53)	24 (18.75)	31 (24.22)	26 (20.31)	2 (1–3)

## Data Availability

Not applicable.
